# Parent Feeding Practices in the Context of Food Insecurity

**DOI:** 10.3390/ijerph18020366

**Published:** 2021-01-06

**Authors:** Katherine R. Arlinghaus, Melissa N. Laska

**Affiliations:** Division of Epidemiology and Community Health, University of Minnesota School of Public Health, Minneapolis, MN 55454, USA; mnlaska@umn.edu

**Keywords:** parent feeding practices, food insecurity, poverty, motherhood, child feeding

## Abstract

The process of feeding is complex and highly dependent on parent, child, social, and environmental factors. Given the rising rates of food insecurity and concomitant poor nutrition and health, the purpose of this article was to outline the important and complex ways in which the context of food insecurity can impact parent feeding practices. Key factors discussed here include the impact of food insecurity on: expectations for motherhood, structural constraints, stress and depression, parents’ perceptions of health and child weight, and intergenerational transmission of parent feeding practices. Future research needs are also identified and discussed.

## 1. Introduction

Food insecurity is associated with poor health and is particularly concerning among children, given that they are growing and developing [1,2]. Food insecurity can also indirectly affect children through its impact on parenting. Parents experiencing food insecurity exhibit greater depressive affect, are quicker to become frustrated with their children, and are less likely to be responsive to child needs [3,4]. As “nutritional gatekeepers”, parents make many decisions regarding what and how much children eat [5]. Food insecurity can influence how parents procure, prepare, and provide food to their children as well as parental goals and behaviors during feeding.

In 2019, the national prevalence of household food insecurity in the United States—defined as not having access by all people at all times to enough food for an active, healthy life—was 10.5% [6]. However, the COVID-19 pandemic dramatically reversed previous declines in food insecurity, and the prevalence of household food insecurity rose dramatically in the Spring of 2020, with reports as high as 38% in late March [7,8,9]. Given this increase, it is critical that parent feeding practices be examined under the context of food insecurity.

Parent feeding practices refer to the specific goal-oriented directives parents engage in when feeding their children and describe the ways in which parents feed their children [10]. Parent feeding practices include behaviors such as restricting food, pressuring children to eat, setting rules about food consumption, monitoring what children eat, providing praise, and modeling eating behaviors [11]. Regarding child food consumption and health outcomes, the way children are fed may be just as important, if not more important, as what and how much food is served [12].

Overall, feeding is a dynamic, reciprocal process in which how parents feed their children depends on child behaviors and characteristics, and child eating behaviors are influenced by parent actions [13,14,15,16,17]. In addition to these person-centered contexts, how parents feed their children can also be influenced by the experience of financial hardship and food insecurity. It is important to better understand these influences to more effectively promote child health and well-being. The goal of this narrative review was to highlight how the context of food insecurity can impact the ways in which parents feed their children in the United States, with a particular emphasis on the association between food insecurity and parent feeding practices. Opportunities for future research and intervention are discussed.

## 2. The Role of Motherhood and Feeding in the Context of Food Insecurity

Child feeding is more than just the provision of fuel. Feeding can be used as a demonstration of love and caring for one’s family, a way to pass along family and cultural traditions to the next generation, and an illustration of empowerment over one’s life [18,19,20,21,22,23]. Socially defined expectations for motherhood also overlap with female identity [24]. Although other individuals can take on the role of feeding children, and recently, greater attention has been drawn to the role of fathers in feeding and child health, mothers are the most common person to prepare food for children [25]. Feeding as a maternal role is constructed and reinforced by society through medical advice, family members and friends, and media [22,24].

Expectations for ideal mothering have been described as having “selfless devotion to their children and following expert advice on how to feed, socialize, and educate their children” [26], and mothers are often blamed by both themselves and society when their children experience adverse health [27]. Given societal pressures, child feeding has become a mechanism through which mothers are critically judged for deviating from social expectations. For example, at a time when breastfeeding is widely promoted as the preferred choice for infant feeding, women who choose to bottle feed often feel the need to justify their choice to prevent being judged as a “bad mother” [28,29]. Experiences of blame and feelings of shame for deviating from social ideals occur among mothers across socioeconomic statuses (SES). However, breastfeeding is disproportionately challenging for mothers of lower SES, and compared to mothers of higher SES, mothers of lower SES return to work sooner after giving birth and are less likely to have work environments that accommodate breastfeeding [30]. Thus, these mothers are more likely to face critical judgement for their feeding decisions, many of which they have been forced to make due to structural constraints they face.

Furthermore, mothers of lower SES face greater public accountability for meeting motherhood expectations than mothers of higher SES [31]. For example, to obtain benefits from food assistance programs, such as the Special Supplemental Nutrition Program for Women, Infants, and Children (WIC), mothers must disclose their feeding practices and mothering choices to strangers. In this way, society makes mothers with fewer resources more accountable for meeting a perceived “mother ideal” than mothers with more resources. Irrespective of a child’s health, being unsure of how she will fulfill her maternal role of feeding her child and/or fearing her child will be hungry is often accompanied by feelings of guilt, shame, or depression [32,33]. Overall, this background is critical to understanding feeding responsibility and child health in the context of food insecurity.

## 3. The Risks of Emphasizing Personal Responsibility

National dialogue about health behavior change has historically been centered on the idea of personal responsibility [34]. Many health behavior change theories, like the Health Belief Model, lack consideration of the influence of economic, social, and cultural contexts on mothers’ ability to make health behavior changes for themselves and their children [35]. In contrast, ecological models explain how individual behaviors are shaped by interactions with and influences from social networks, institutional structures, communities, and large-scale societal factors, like public policy [36,37].

This emphasis on structural constraints helps more accurately frame child health and avoids exclusively blaming parents for poor outcomes. Because how a mother feeds her child can be a source of identity and pride, putting an emphasis on personal responsibility can be detrimental—particularly when overlooking factors like material hardship, stigma, and structural racism. For example, parents of children with obesity face substantial stigma [38,39,40], and when internalized, stigma has been associated with counter-productive parenting including fewer family meals, encouragement of dieting, restrictive feeding practices, and an avoidance of situations in which parents are likely to be blamed like well-child exams [41,42,43]. Thus, in trying to improve children’s health by working with parents, clinicians and researchers face the risk of worsening parent and child health through stigmatizing behaviors and messages, including those of personal responsibility, even when unintentional.

## 4. Structural Constraints Impact Parent Feeding Practices

From a socioecological perspective, structural constraints such as proximity to healthy food, time, and the cost of food are considered important influences of how parents feed their children. Food scarcity reduces parental ability to control access to unhealthy foods, as food choices become restricted by limited financial resources [44,45]. Eating healthfully on a limited budget can be a challenging, time-intensive process. Those experiencing food insecurity often also have significant additional time constraints. For example, compared to those at higher income levels, families experiencing food insecurity are more likely to be those of single parents [46,47]., Single parents, by definition, have more responsibility as an individual and thus more time constraints, compared to parents in two-parent households. Given this context, they are less likely to have the ability to be as involved in healthy meal planning and food preparation with and/or for their children [48].

The development of food preferences, which involves repeated exposure to novel foods, may also be altered under the context of food insecurity or financial hardship. For example, previous research has shown that more than 15 exposures to a food may be needed before a child develops a liking [49]. However, mothers experiencing food insecurity often do not feel able to offer a food that is unlikely to be eaten, especially if it is a more expensive and perishable food like a fresh fruit or vegetable [50]. As such, the lower consumption of fruits and vegetables among children and families experiencing food insecurity may be due to both an effort to prevent food waste and the higher cost of these foods [50,51].

One strategy to help facilitate the introduction of novel foods to children is to serve all family members the same meal [52]. Whether aimed at ensuring children eat, mitigating conflict at the meal, or minimizing food waste, parents often provide an alternative meal consisting of preferred foods to children who engage in fussy eating behaviors [51,53]. This practice reduces the likelihood that children will try novel foods and may reinforce picky eating [52]. While feeding children who exhibit fussy eating can be challenging for all parents, how parents respond to fussy eating behaviors likely differs based on food security status. Among low-income families, parents experiencing food insecurity are less likely to provide alternative meals than those experiencing food security [51]. Among low-income families that do provide alternative meals for picky eaters, the alternative meals are quick to prepare and rarely contain fruits or vegetables [54]. This indicates that the difference in alternative meal provision by food security status is likely driven by a difference in available resources rather than a difference in intention to engage in recommended feeding practices.

Under conditions of resource scarcity, parents may monitor and control child food access more. Controlling feeding practices such as restriction (limiting child access to “unhealthy” foods) and pressure to eat (“clean your plate”) are thought to disrupt children’s innate ability to self-regulate how much they eat and are generally associated with problematic eating behaviors and obesity [55]. Although restriction and pressure to eat have primarily been studied in relation to child weight and obesity risk [56], parents may engage in restrictive feeding if the family is experiencing food insecurity as a way to conserve resources [57]. Similarly, parents may pressure their children to eat available food because they are unsure of when they will have food again and/or so that food is not wasted.

Research regarding the relationship between food insecurity and controlling feeding practices has produced mixed findings. Consistent with the idea that restriction is associated with less healthy self-regulation and obesity, one study found greater restrictive feeding, greater eating in the absence of hunger, and a higher prevalence of child obesity among households experiencing food insecurity, compared to those with food security [58]. While some research has found food insecurity to be associated with greater restrictive feeding practices [57,58,59,60], others have found no association [61]. Evidence regarding the relationship between food insecurity and pressure to eat has also been mixed. Food insecurity has been associated with greater pressure to eat in some studies [59,62,63,64] and no association in others [58,61]. In food secure households, pressure to eat has had a positive association with child vegetable consumption but not in households experiencing food insecurity [65].

Overall, most of the existing literature has focused on adverse parent feeding practices in the context of food insecurity, and very little is known about the positive traits of parent feeding in this context. Notably, available data, although limited, suggest no relationship between food security status and favorable feeding practices like modeling [66] and positive reinforcement for healthy consumption [62,66]. These findings are important because they illustrate that despite experiencing food insecurity, parents are able to engage in beneficial parent feeding practices at the same level as those with food security.

Further research is needed, particularly prospective longitudinal studies that comprehensively investigate both favorable and unfavorable feeding practices, to clarify the role of parent feeding practices in the context of food insecurity. A better understanding of how parents who experience food insecurity are able to engage in favorable parent feeding practices can help inform what support may be most beneficial for families with food insecurity. Despite its limitations, however, the existing literature clearly indicates that food insecurity can not only influence the availability of food but also the way in which parents feed their children.

## 5. Stress and Mental Health as Important Mediators

Consistent with the Family Stress Theory, the experience of food insecurity can also influence parent feeding through increased stress and/or depression [67]. Maternal depression and food insecurity are positively associated [46,68], and having depression or high stress levels can interfere with a parent’s ability to appropriately respond to her child during feeding [69,70,71,72]. Furthermore, limited research suggests that high levels of stress and depression are associated with more controlling feeding practices like greater pressure to eat and the use of food as a reward or bribe [70,73].

Research explicitly examining the relationships between maternal stress and depression, food insecurity, and parent feeding practices is sparse. However, evidence among low-income families suggests that the relationship between maternal stress and depression and parent feeding practices may differ by food security status. Among families with food insecurity, maternal stress or a depressed mood earlier in the day was associated with greater restriction but not with pressure to eat [57]. Conversely, among food secure families, maternal stress earlier in the day was associated with greater pressure to eat, but not with restriction [57,74].

Notably, the relationship between maternal depression and food insecurity can be bidirectional [47,75]. In addition to food insecurity leading to depression, depression has been found to lead to future food insecurity. Economic hardship is related to other forms of adversity such as housing instability, martial conflict, and exposure to violence, all of which can be extremely stressful and associated with depression [76,77]. Depression and stress can impact energy levels to cook and shop and may reduce mothers’ ability to navigate assistance programs that help alleviate food insecurity [68]. For example, higher rates of depression are associated with reduced WIC and Supplemental Nutrition Assistance Program (SNAP) participation [46,78]. Further research is needed to better understand the complex relationship between maternal stress and depression, food insecurity, and parent feeding practices.

## 6. Intergenerational Impact of Food Insecurity on Parent Feeding Practices

Experiencing food insecurity as a child is highly correlated to experiencing food insecurity as an adult [79]. Adverse outcomes from the intergenerational transmission of food insecurity are likely to disproportionately affect communities of color, as Black Americans have higher rates of downward income mobility than White Americans, due to structural racism and related factors [80,81]. Experiences of chronic food insecurity can influence how children learn to acquire and prepare food and may impact family beliefs and values [82]. Through experiences of childhood food insecurity, children learn food management strategies that prepare them to be able to provide food for their families as an adult [83,84].In food insecure households, a “healthful” diet may be based on having a sufficient quantity of food rather than being focused on the nutritional quality of the food provided [65]. These experiences and values are likely to have long-term psychosocial impacts that influence parent feeding behaviors. For example, parents who experienced food insecurity as a child report being highly motivated to prioritize feeding their children over all other necessities [83].

Consistent with the life course perspective [85], childhood memories of eating influence parent feeding practices, irrespective of current food security status [86,87]. Many low-income parents describe wanting to give children what they wished they had as a child and to protect their children from feelings of deprivation [88,89]. In particular, parents who experienced early food insecurity report finding it difficult to say no to children’s requests for food, especially when it is plentiful at the beginning of the month, and often the food requested is not of high nutritional quality [83,88]. Previous research has shown that consumption of sweets and soda is higher among children of parents who remember experiencing food insecurity [90]. Similarly, parents with past experiences of food insecurity are less likely to monitor their children’s consumption of sweets and snack foods [91]. These practices are consistent with an indulgent feeding style [92]. Indulgent feeding is common among low-income families, particularly low-income Hispanic families, and is associated with obesity [93,94].

Food insecurity as a child has also been associated with parent perceptions that their children do not weigh as much as they should [90], and parents experiencing food insecurity are more likely to inaccurately perceive their healthy weight children as being underweight [95]. This may lead parents to pressure their child to eat and to not set limits on foods high in calories which lead to excess weight gain. Importantly, among low-income households, excess weight is often only perceived as a health issue if it interferes with children’s ability to be active or results in being teased [96]. Understanding these perceptions is critical to informing intervention recruitment and framing, given that using obesity prevention as motivation to improve healthy feeding practices may not resonate particularly well with low-income parents and/or those experiencing food insecurity. Parents may be more motivated to participate in parent feeding interventions aimed at preventing diabetes which has been described as particularly concerning [86].

Lastly, experiencing food insecurity may incite maladaptive eating behaviors. Binge eating at the beginning of a benefit cycle or when food is readily available is commonly reported among families experiencing food insecurity [83]. This period is often followed by a period of restriction in which food is less plentiful, which results in a “feast–famine” cycle of eating that is commonly exacerbated by the timing of SNAP assistance [97]. While less research has been conducted among pediatric populations and longitudinal research is lacking, previous studies have shown a consistent cross-sectional association between food insecurity and greater eating disorder pathology [98]. Among women with food insecurity, binge eating and emotional eating behaviors distinguished women with obesity from those with healthy weight [99]. This indicates that these maladaptive eating behaviors may be one mechanism to explain the paradoxical relationship between food insecurity and obesity.

Learned binge eating behaviors through childhood experiences of food insecurity could continue into adulthood, even in instances in which food is no longer scarce. Children whose mothers engage in binge eating are more likely to also engage in this type of behavior [100,101]. Mothers who engage in binge eating may be more likely to use controlling feeding practices [102]. Thus, past experiences of food insecurity and resulting maladaptive eating behaviors can be transmitted across generations. Further research is needed to better understand how experiencing food insecurity impacts individuals’ relationships with food and how this may impact parent feeding practices.

## 7. Conclusions

There are many gaps in evidence regarding food insecurity and parent feeding. However, existing evidence indicates that the context of food insecurity has important implications for how parents feed their children. Given the evidence that experiencing food insecurity as a child can influence parent feeding practices, interventions aimed at improving parent feeding behaviors are unlikely to be effective if they do not address these antecedents of parent feeding practices. Childhood experiences, family and cultural values, social roles of motherhood, and perceptions of health are integral considerations to understanding structural and personal constraints and facilitators to healthy feeding behaviors.

Interventions can be informed from a better understanding of factors that differentiate individuals who emerge from the experience of child food insecurity with resiliency from those who do not. Historically, the parent feeding literature has been disproportionately focused on unfavorable, controlling parent feeding practices. This is in part due to the omittance of favorable feeding practices in the original and most widely used questionnaires to assess parent feeding [103]. The skewed amount of evidence on what *not to do* compared on what *to do* has likely delayed the development of effective interventions. Currently, most of the literature regarding parent feeding practices and food insecurity is again focused on unfavorable feeding practices.

The infancy of the literature regarding parent feeding practices and food insecurity presents an important opportunity to remediate this by more equitable investigation of feeding practices associated with both favorable and unfavorable health outcomes. Taking a positive deviance approach to understanding the relationship between food insecurity and parent feeding may be an important strategy to empower and aid families to achieve improved health. In contrast to deficit-based approaches in which what did not work or was harmful is identified, a positive deviance approach focuses on what was helpful in the past. Specifically, a positive deviance approach identifies “social deviants” who have healthy outcomes despite facing the same challenges and having no more resources than their peers [104]. A social deviance approach to childhood obesity is starting to become more common [105,106]. This work has found self-efficacy to be a salient characteristic of positive deviant families who were able to reduce child adiposity [106]. To our knowledge, a social deviance approach has not been used to identify positive deviants among families experiencing food insecurity who have been able to improve child eating behaviors. Studying the actions and attributes of parents who engage in health promoting feeding practices despite experiencing food insecurity, as well as their structural supports and the larger context of their lives, may identify important intervention targets to improve parent feeding under the context of food insecurity. Importantly, the identification of families experiencing food insecurity who are able to improve child eating behaviors requires the opportunity to do so through intervention supports. As such, this approach may help the field progress past observational work into intervention studies more quickly.

Given the long-lasting negative physical and mental health associations with food insecurity, it is critical that existing food assistance programs are protected and that innovative ideas for additional mechanisms to reduce food insecurity are supported. It is likely that reductions in food insecurity will require a cross-disciplinary, food systems approach [107]. Programs like WIC and SNAP as well as minimum wage and affordable housing policies have been shown to alleviate food insecurity [108]. Similarly, the Child and Adult Care Food Program (CACFP) and National School Lunch and Breakfast Programs are key safety nets for families. While federal policy has enabled these programs to continue during the pandemic, a lack of capacity among institutions that facilitate these programs has weakened implementation and reach [109,110]. For example, due to the pandemic, many early child-care and education programs that provide free meals through the CACFP had to close or terminate some staff positions, which resulted in a lack of capacity to facilitate meal distribution programs [109]. In Connecticut, for instance, about 80% of centers participating in CACFP closed, and only 15% were able to continue providing CACFP-reimbursable meals [109].

Financial and food assistance can help ensure parents have healthy foods to feed their children. Given that parent feeding is more than the provision of food, it is also important that assistance programs address how parents feed their children. In addition to food assistance, programs like WIC and SNAP also include education components that offer intervention opportunities to address parent feeding practices. Building from WIC’s success at increasing breastfeeding rates and helping parents more accurately respond to infant cues, it has been recommended that more in depth responsive feeding interventions be standardized through WIC services [111,112]. Although experimental research on parent feeding is relatively scarce, existing interventions have demonstrated improvements in parent feeding practices [112,113,114]. In particular, an Australian parenting intervention focused on increasing healthy food exposure, responsive feeding, and positive parenting resulted in improvements in responsive feeding that were sustained five years later [115]. Using these interventions as models that could be integrated into existing food assistance and education programs may be an important strategy to simultaneously address both what and how parents feed their children. This type of intervention work has the capability to provide direct evidence regarding how food insecurity may shape parent feeding practices.

Poverty is arguably the most prominent feature of food insecurity. Research specifically among communities of color is of high urgency, as the additional social and structural constraints that lead these families to have an increased risk of food insecurity have also led to these families to be disproportionately impacted by the pandemic [116,117]. Racial discrimination is associated with substantial increases in an individual’s risk of food insecurity [118]. As such, it is imperative that moving forward, parent feeding interventions consider and address both structural racism and food insecurity. This is particularly pertinent as most feeding interventions have been conducted among White, middle–upper class families [112].

To our knowledge, existing interventions aimed at improving parent feeding practices have not specifically addressed parenting in the distinct context of food insecurity, though some have been developed for lower-income groups. While the literature on the direct relationship between food insecurity and parent feeding practices is limited, existing evidence suggests food insecurity likely impacts what and how parents feed their children. The COVID pandemic has specifically highlighted the influence food insecurity can have on parent feeding practices. Compared to families with food security, parents experiencing food insecurity increased the frequency at which they pressured their children to eat during the COVID pandemic [64]. While the long-term impact of changes in parent feeding practices during this time are unknown, changes in parent feeding practices in response to growing food insecurity due to the COVID pandemic are demonstrative of the nuanced ways in which this period may have lasting implications for child health. It will be important to monitor how food insecurity may impact parent feeding practices moving forward and develop and/or adapt parent feeding interventions to meet the needs of families experiencing food insecurity.

## Data Availability

Not applicable.
